# The Combined Analysis of GC-IMS and GC-MS Reveals the Differences in Volatile Flavor Compounds between Yak and Cattle-Yak Meat

**DOI:** 10.3390/foods13152364

**Published:** 2024-07-26

**Authors:** Ben Zhang, Mengli Cao, Xingdong Wang, Shaoke Guo, Ziqiang Ding, Yandong Kang, Liyan Hu, Lin Xiong, Jie Pei, Yi Ma, Xian Guo

**Affiliations:** 1 Key Laboratory of Yak Breeding of Gansu Province, Lanzhou Institute of Husbandry and Pharmaceutical Sciences, Chinese Academy of Agricultural Sciences, Lanzhou 730050, China; zhangbencaas@163.com (B.Z.); caomengliaaa@163.com (M.C.); wxd17339929758@163.com (X.W.); gsk1125@163.com (S.G.); dingziqiang1997@163.com (Z.D.); kangyandong0901@163.com (Y.K.); huliyan2020@163.com (L.H.); xionglin@caas.cn (L.X.); peijie@caas.cn (J.P.); 2 Key Laboratory of Animal Genetics and Breeding on Tibetan Plateau, Ministry of Agriculture and Rural Affairs, Lanzhou 730050, China; 3 Institute of Animal Husbandry and Veterinary Science, Tianjin Academy of Agriculture Sciences, Tianjin 300381, China

**Keywords:** yak, cattle-yak, GC-IMS, GC-MS, VOCs, metabolites

## Abstract

In order to investigate the composition and differences in volatile organic compounds (VOCs) in yak and cattle-yak meat and determine the key metabolites and metabolic pathways related to flavor formation. In this study, the VOCs and non-volatile metabolites in Longissimus dorsi muscle of two groups of samples were detected and analyzed by gas chromatography–ion migration spectrometry (GC-IMS) and gas chromatography–mass spectrometry (GC-MS). The results showed that 31 VOCs were identified by GC-IMS, including 5 alcohols, 5 ketones, 5 esters, 3 aldehydes, 2 furans, 2 hydrocarbons, 1 amine, 1 acid, 1 thiazole, 1 pyrazine, and 5 others. Most of them were alcohols, ketones, esters, and aldehydes. A total of 75 non-volatile metabolites with significant differences were obtained by GC-MS screening, among which amino acid contents such as serine, glycine, phenylalanine, and aspartic acid were significantly up-regulated in cattle-yak, and glutamic acid and tyrosine were significantly up-regulated in yak. The non-volatile differential metabolites in the two groups were significantly enriched in the metabolic pathways of arginine biosynthesis and oxidative phosphorylation. By combining GC-IMS and GC-MS, this study comprehensively and intuitively reflected the differences in VOCs between yak and cattle-yak meat, and clarified the metabolomic reasons for the differences in VOCs, so as to provide a theoretical basis for meat quality improvement.

## 1. Introduction

Yak (*Bos grunniens*) is a landmark species in the high altitude area of the Qinghai-Tibet Plateau in China, living in the natural grassland at an altitude of 2700~5000 m, and has a high adaptability to the low-temperature and low-oxygen environment in the plateau area [1]. China has the largest population of yaks in the world, with 14 million heads, accounting for 95% of the global yak population [2]. Yaks are essential to the daily lives of the locals since they can provide a variety of goods, including meat, milk, wool, and leather [3]. Over the years, yak meat has been more and more favored by people because of its green, pollution-free, high protein and mineral content; low fat content; dark meat color; coarse muscle fiber; unique flavor; and other advantages [4]. Cattle-yak is an interspecific hybrid between yak and cattle, with the female of the F1 generation having reproductive ability, while the male is sterile [5]. Cattle-yak shows obvious heterosis in growth rate, drought tolerance, and disease resistance, and the meat produced by cattle-yak has similar quality characteristics to yak meat, especially in game meat flavor [5,6]. However, in recent years, consumers have become less appreciative of ordinary meat, and meat with high quality or unique flavor has become more popular [7]. Therefore, attention should be paid to the local characteristic beef breeds, their meat quality should be evaluated, and the formation mechanism of their unique flavor substances should be studied. At present, most of the comparative studies on yak and cattle-yak focus on genetic differences, and there is a lack of comparison on the differences in meat flavor substances between breeds [6]. Flavor quality plays a vital role in the sensory characteristics of food, and is an important index to evaluate the nutritional value and freshness of food, and directly affects consumers’ purchase intention and acceptance degree [8]. Therefore, it is necessary to investigate the differences in VOCs between yak and cattle-yak meat.

Sensory analysis and instrumental analysis are two commonly used methods for VOC analysis in food, among which instrumental analysis is more practical for the identification of VOCs at a molecular level [9]. Gas chromatogram–ion migration spectrometry (GC-IMS) is a new type of hot gas phase separation detection technology, which combines the high separation ability of GC with the fast response ability of IMS, with the advantages of no sample pretreatment, fast analysis time, low detection limit, and simple operation [9,10]. GC-IMS is widely applied in drug testing, disease surveillance, and other fields, especially the analysis of food flavor [8]. Nevertheless, although GC-IMS exhibits high sensitivity and rapid reaction speed, it is only capable of detecting ionic compounds, such as alcohols, aldehydes, and olefins, which possess a higher proton affinity than water [9]. In addition, because the current database of gas phase retention index and database of the migration time used for qualitative analysis in GC-IMS is not complete enough, some volatile substances cannot be identified [11]. Gas chromatography–mass spectrometry (GC-MS) based on metabolomics can act as an effective technique to isolate and identify complex volatile compounds in the qualitative and quantitative detection of volatile components [12]. It has the advantages of high resolution and high sensitivity, and is regarded as the gold standard of food flavor analysis [12,13]. In contrast to GC-IMS, GC-MS analysis is based on detailed information on compounds provided by a standard reference database [14]. However, GC-MS is not sensitive to lower levels of volatile flavor substances, which can easily be neglected [15].

In recent years, the combination of GC-IMS and GC-MS has been successfully used for volatile flavor analysis of tea [16], aquatic products [12], and corn [17], which can not only isolate and identify a wider range of volatile compounds but also provide a more comprehensive and diversified food flavor profile. However, there are few reports on the difference in VOCs in yak and cattle-yak meat by combining GC-IMS and GC-MS, which provides a new insight for this study.

This study aims to combine GC-IMS and GC-TOF-MS technology to determine the VOCs and metabolic profiles in yak and cattle-yak meat, explore the precursor substances and metabolic pathways of VOCs in meat, provide a theoretical basis for exploring the regulatory mechanism of the formation of VOCs in yak and cattle-yak meat, and also point out a direction for the improvement of meat quality.

## 2. Materials and Methods

### 2.1. Ethics Statement

All the animal procedures were carried out in accordance with the guidelines of the Chinese Animal Protection Commission and the Ministry of Agriculture of the People’s Republic of China. The yak and cattle-yak treatment procedures were approved by the Animal Care and Use Committee of Lanzhou Institute of Husbandry and Pharmaceutical Sciences, Chinese Academy of Agricultural Sciences (Permit No: SYXK-2014-0002).

### 2.2. Sample Collection

Six yaks (Ys) and six cattle-yaks (CYs), both male and in good condition, with similar weight and age of 4.5 years old, were selected from Hezuo City, Gannan Tibetan Autonomous Prefecture (35.01° N, 102.92° E). After slaughter, fresh longissimus dorsi muscles from the left side were taken for the determination of VOCs and metabolites. At the same time, part of the meat samples were packaged in self-sealing bags and transported back to the laboratory at 4 °C for the determination of protein, fat, and mineral elements.

### 2.3. Nutrient Composition Determination

The nutrient composition of longissimus dorsi muscles was analyzed by the Chinese recommended standard. According to GB/T 5009.5-2016 [6], the protein content was determined by the Kjeldahl nitrogen method. The fat content was determined by the Soxhlet extraction method according to GB/T 5009.6-2016. The P content was determined according to GB/T 5009.87-2016. The contents of Ca and Fe were determined according to GB/T 5009.268-2016. 

### 2.4. GC-IMS Determination

#### 2.4.1. Sample Processing

The crushed longissimus dorsi muscle samples of the yak and cattle-yak were accurately weighed by 2 g each and placed in a 20 mL headspace bottle for analysis by GC-IMS (FlavorSpec^®^, G.A.S. Dortmund, Germany).

#### 2.4.2. GC-IMS Conditions

Column type: FS-SE-54-CB-1 (15 m ID: 0.53 mm); analysis time: 20 min; column temperature: 60 °C; carrier gas/drift gas: N_2_; IMS temperature: 45 °C. Headspace automatic injection, injection volume: 500 μL; incubation time: 15 min; incubation temperature: 60 °C; injection needle temperature: 80 °C; incubation speed: 500 r/min. The initial carrier gas flow rate is 2 mL/min. The flow rate remained at 2 mL/min within 0~2 min, and the carrier gas flow rate increased linearly from 2 mL/min to 100 mL/min within 2~20 min. The drift gas flow rate is 150 mL/min.

#### 2.4.3. GC-IMS Statistical Analysis

The NIST database and IMS database built in the GC-IMS instrument were used for the qualitative analysis of VOCs. The Reporter and Gallery plot plug-ins in the Laboratory Analytical Viewer (LAV, G.A.S. Dortmund, Germany) were used to construct the 3D and 2D differential profiles and fingerprints of VOCs.

### 2.5. Metabolomics Analysis

#### 2.5.1. Extraction of Metabolites

A total of 50 ± 1 mg of the sample was put into a 2 mL EP tube, 500 μL of pre-cooled extract (methanol to chloroform ratio = 3:1), containing internal standard (adonitol, 0.5 mg/mL stock) was added, and vortexed for 30 s. We added a steel ball, put it in a 40 Hz grinder for 4 min, and performed ice water bath ultrasound for 5 min (repeat 3 times). The sample was centrifuged at 4 °C at 12,000 rpm (centrifugal force 13,800× *g*, radius 8.6 cm) for 15 min. We carefully removed 200 μL supernatant into 1.5 mL EP tube, and took 80 μL of each sample and mixed into the QC (quality control) sample. After evaporation in a vacuum concentrator, 30 μL Methoxyamination hydrochloride (20 mg/mL in pyridine) was added, incubated at 80 °C for 30 min, and then derivatized with 40 μL BSTFA reagent (1% TMCS, *v*/*v*) at 70 °C for 1.5 h. The sample was gradually cooled to room temperature. In total, 5 μL FAMEs (chloroform) was added to the QC sample. Subsequently, gas chromatography combined with time-of-flight mass spectrometry (GC-TOF-MS) was used to analyze all the samples.

#### 2.5.2. GC-TOF-MS Conditions

The GC-TOF-MS analysis was conducted via an Agilent 7890 (Agilent Technologies, Wilmington, Delaware, USA) gas chromatograph coupled with a time-of-flight mass spectrometer. The system employed a DB-5MS capillary column for its operation. In total, 1 μL aliquot of the sample was injected in splitless mode. The carrier gas was helium, the front inlet purge flow was 3 mL min^−1^, and the gas flow rate in the column was 1 mL min^−1^. The initial temperature was maintained at 50 °C for 1 min, then increased to 310 °C at a rate of 10 °C min^−1^, and then maintained at 310 °C for 8 min. The temperatures of the injection, transfer line, and ion source were set to 280 °C, 280 °C, and 250 °C, respectively. In the electron impact mode, the energy was −70 eV. After a solvent delay of 6.4 min, mass spectrometry data were obtained in full scan mode at a rate of 12.5 spectra per second, with the m/z range of 50–500.

#### 2.5.3. Analysis of GC-TOF-MS Data

The Chroma TOF software (V 4.3x, LECO, San Jose, USA) was used for peak extraction, baseline correction, deconvolution, peak integration, peak alignment, and other analysis of the mass spectrum data. The LECO-Fiehn Rtx5 database was used in the qualitative work, including mass spectrum matching and retention time index matching. Finally, the peaks with detection rates below 50% or RSD > 30% in the QC samples were removed. The SIMCA software (version 14.0) was used for multivariate statistical analysis, including principal component analysis (PCA) and orthogonal–partial least squares discriminant analysis (OPLS-DA). Differential metabolites were screened by the criteria of variable importance in projection (VIP) > 1 and *p* < 0.05, and metabolic pathway annotations were performed through the KEGG database. Enrichment analysis was further used to find the key pathways with the highest correlation with differential metabolites.

### 2.6. Data Processing

The experimental data were preliminarily summarized by Excel 2010, and an independent sample *T*-test was conducted by IBM SPSS 26.0 (IBM, Armonk, NY, USA). *p* < 0.05 was considered statistically significant. All the trials consisted of 6 biological replicates per group, and the results were presented as mean ± standard deviation (SD).

## 3. Results and Discussion

### 3.1. Nutrient Composition Analysis

As shown in Table 1, the protein content in Y was 24.34%, which was significantly higher than that in CY (*p* < 0.05). Protein is an essential substance for various metabolic activities of the human body and it also plays an important role in food processing because of its significant influence on the nutritional value and technical functional attributes of food [18]. The high content of protein in yak meat is a crucial raw material for making functional food such as protein powder and protein powder extracted from yak meat can strengthen muscle and enhance immunity [19]. Tenderness is the most important characteristic that determines consumer acceptance and satisfaction [20]. The fat content in meat is positively correlated with the tenderness of meat, which has a great influence on the quality of meat [21]. The high content of fat in Y contributed to its great meat tenderness. The Fe content in Y was significantly higher than that in CY (*p* < 0.05). Fe is an indispensable mineral element of hemoglobin and myoglobin. When the supply of Fe is insufficient, the human body will have a certain degree of anemia [19]. Yak meat, as a food with rich iron content, has the potential to prevent iron deficiency anemia. Above all, compared with cattle-yak, yak meat is rich in protein, fat, and mineral elements, which is worthy of further development.

### 3.2. GC-IMS Analysis

#### 3.2.1. GC-IMS Topographic Plot Analysis

**Figure 1:**
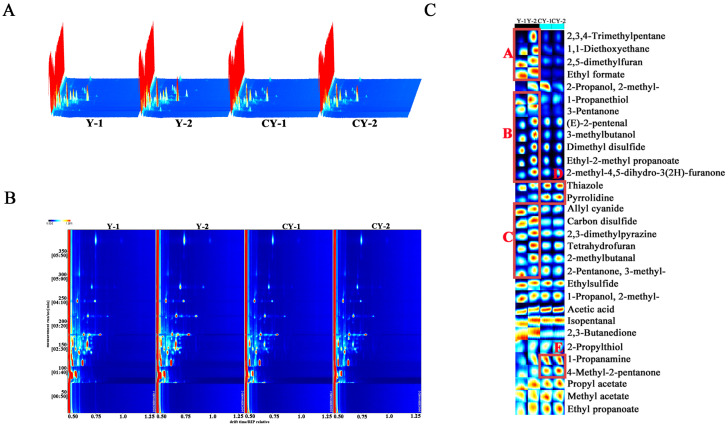
(**A**) Three-dimensional spectrogram of Y and CY. (**B**) Two-dimensional top view of Y and CY. (**C**) Fingerprints of VOCs in Y and CY.

The GC-IMS detection results of VOCs in Y and CY are shown in Figure 1A. The data were visually represented by three-dimensional (3D) spectrograms. In Figure 1A, the X-axis is the ion drift time, the Y-axis is the retention time of the gas chromatograph, and the Z-axis is the ion peak intensity, each peak corresponding to a specific volatile compound, whose intensity is represented by a color. It can be seen that the peak signal distribution of Y and CY were similar, but there were some differences in the peak signal intensity of each group of samples, indicating that there were differences in the contents of VOCs in the two groups of samples.

Due to the roughness of the 3D spectrum, for the convenience of observation, a two-dimensional (2D) top view was used to compare the differences in VOCs in Y and CY. Figure 1B shows the GC-IMS 2D plot of the VOCs in Y and CY, and the whole 2D plot represents all the VOCs of the samples. The red vertical line at 0.50 of the X-axis represents the reactive ion peak (RIP, normalized), and each point on the right of RIP represents the volatile substance isolated from the samples. The content of the substance is qualitatively indicated by the color of the point, with red indicating a higher content and white indicating a lower content. It can be seen that the signal peaks of most VOCs appeared within the retention time of 50–300 s and the migration time of 0.50–0.75 ms, and the compounds were more concentrated within the retention time of 50–200 s, and more dispersed within 200–300 s. This may be mainly due to the differences in the polarity of different compounds, resulting in different retention times of polar and non-polar compounds after passing through the non-polar column [22].

#### 3.2.2. Fingerprint and Qualitative Analysis of VOCs

Topographic plots can visually show the changing trend in VOCs; however, it is difficult to make a correct judgment of the closely linked substances on the plot, and this problem can be solved by using fingerprint analysis [23]. The Gallery Plot plug-in of the LAV software (https://www.gas-dortmund.de/Products/Software/Laboratory-Analytical-Viewer/1_463.html, accessed on 24 July 2024) was used to select material signals in Y and CY sample spectra to form fingerprints. As shown in Figure 1C, the variation pattern and relative content of VOCs in the Y and CY samples can be observed more specifically and intuitively, with each column showing the entire signal intensity of one sample and each row showing the same VOCs in different samples. In addition, the color represented the signal intensity (compound content) of VOCs, with low intensity indicated by white and high intensity by red, with darker red indicating higher signal intensity. It can be observed that some substances only had the highest content in one sample, which was higher than that in other samples, and can be used to distinguish the differences between the different samples.

Four substances can be observed in the A region, namely 2,3,4-trimethylpentane, 1, 1-diethoxyethane, 2,5-dimethylfuran, and ethyl formate. Seven substances are in the B region, namely 1-propanethiol, 3-pentanone, (E)-2-pentenal, 3-methylbutanol, dimethyl disulfide, ethyl-2-methyl propanoate, and 2-methyl-4,5-dihydro-3(2H)-furanone. Six substances in the C region, namely allyl cyanide, carbon disulfide, 2,3-dimethylpyrazine, tetrahydrofuran, 2-methylbutanal, and 2,3-butanedione, were the highest in Y. Thiazole, pyrrolidine in region D and 1-propanamine, 4-methyl-2-pentanone in region E have the highest content in CY. 

In the GC × IMS library search, a total of 31 VOCs were identified, including 5 alcohols, 5 ketones, 5 esters, 3 aldehydes, 2 furans, 2 hydrocarbons, 1 amine, 1 acid, 1 thiazole, 1 pyrazine, and 5 others, among which alcohols, ketones, and esters were the majority, as shown in Table 2. Aldehydes, alcohols, ketones, and esters are considered key aroma compounds due to their low odor thresholds and large contribution to odor [24].

Lipid oxidation, precursor degradation, and Maillard reaction are the three main ways to produce VOCs such as aldehydes, alcohols, and ketones [25]. Most aldehydes are produced by the oxidation of unsaturated fatty acids, and very few are produced by the Maillard reaction [26]. Due to their strong aroma and low odor threshold characteristics, aldehydes are considered to contribute significantly to the flavor of meat products [12,23]. 2-methylbutanal is a highly volatile methyl-branched aldehyde, which can be produced by the metabolic degradation of fat and carbohydrate, and can also be produced by the decomposition of protein in meat [14]. The high content of 2-methylbutanal in Y made the yak meat have a fruity and fermented aroma. Most aldehydes have a characteristic fatty aroma at low levels, but when levels are above a certain threshold, they produce rancidity or other odors [23]. Alkenals mainly existed with trans-forms with ten carbon or less, among which (E)-2-pentenal gave yak meat its fatty aroma [27].

The oxidation of unsaturated fatty acids and the degradation of amino acids are the main ways to produce ketones [9]. The difference in flavor is mainly due to the differences in quality and quantity of carbonyl compounds, which play a coordinating role in the formation of the overall aroma of meat [23]. Therefore, ketones have an important effect on the formation of meat flavor. Ketones have a high threshold, much higher than other aldehydes, which are stable in nature and often have floral, creamy, and fruity characteristics [8,12]. The results showed that the contents of 2,3-butanedione, 2-methyl-4,5-dihydro-3(2H)-furanone, and 3-Pentanone in Y were relatively high. 2,3-butanedione was a by-product of the Maillard reaction, and because of its low odor threshold, it was likely to be an important contributor to providing buttery flavor to yak meat [25]. 2-methyl-4, 5-Dihydro-3 (2H)-furanone was a fragrance and aroma component widely existing in nature, giving yak meat flavor and sweetness. It was found that the high content of 4-methyl-2-pentanone in CY gave the cattle-yak meat a pleasant keto-like odor [28].

Alcohols are flavor substances that are produced from polyunsaturated fatty acids as precursors by lactose fermentation, amino acid metabolism, or aldehyde reduction [29]. Most alcohols have pleasant odors such as sweet, fresh, fruity, vegetable, and floral, which increase the volatile flavor of meat products [8]. It was found that there were high contents of 1-Propanethiol and 3-methylbutanol in Y. 3-methylbutanol has a malty aroma and is derived mainly from the Streker degradation of leucine or isoleucine in the Maillard reaction [9]. Due to the high threshold of alcohols, in general, alcohols have little effect on the volatile flavor of yak meat, and only a small amount of alcohols with higher contents have a certain effect on its volatile flavor [8]. It can be concluded that alcohols have less significant effects on the formation of volatile flavor than aldehydes, but they have a synergistic effect on the overall volatile flavor of meat.

Esters are usually formed by the esterification of alcohols and acids [15]. Esters are volatile flavor components commonly found in foods, and most of these compounds, due to their low odor threshold, greatly contribute to providing the desired fruity aroma to meat [23]. Studies have shown that esters containing short-chain fatty acids produce sweet or fruity flavors to a certain extent, while esters containing long-chain fatty acids produce fatty flavors [30]. For example, Ethylformate presented a pleasant, ethereal, diffuse, warm fruity taste, and its content was highest in yak meat and lowest in cattle-yak meat. Ethyl-2-methylpropanoate is an alcohol-like flavor substance with osmanthus fragrance and floral fragrance, which was the highest content in yak meat.

### 3.3. GC-TOF-MS Analysis

#### 3.3.1. Qualitative Analysis of Metabolites

**Figure 2:**
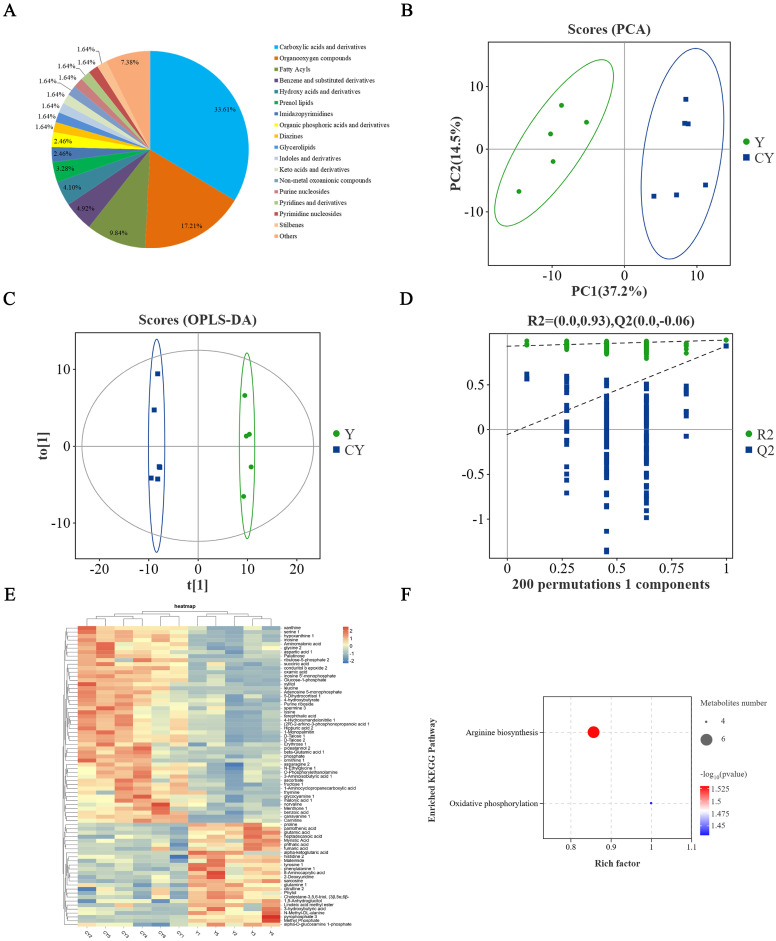
(**A**) Pie chart of the number of different types of 122 non−volatile metabolites in yak and cattle−yak meat. (**B**) PCA model. (**C**) OPLS−DA model. (**D**) Permutations test plot of OPLS−DA model. (**E**) Heatmap of differential metabolites. (**F**) Pathway enrichment of differential metabolites.

The QC samples were prepared by mixing the samples and were used to analyze the repeatability of the samples under the same processing method. The total ion chromatography (TIC) of the QC samples detected by mass spectrometry is shown in Figure S1. The peak retention time and peak area overlap of the QC samples were very good, indicating that the test instrument was stable and the test data were reliable, which can be used for the next analysis. The TIC of the yak and cattle-yak meat samples are shown in Figure S2. Under this detection condition, the shape and distribution of the peaks were relatively uniform, and the chromatographic peaks were mainly concentrated within 30 min. After baseline filtration, peak identification, integration, and retention time correction, a total of 122 metabolites were identified (Table S1). Figure 2A showed the classification of metabolites, which can be divided into 18 categories, among which carboxylic acids and derivatives (41, 33.61%), organooxygen compounds (21, 17.21%), and fatty acyls (12, 9.84%) were the three main metabolites.

#### 3.3.2. Multivariate Statistical Analysis

Metabolomics data have multi-dimensional characteristics, and the variables are highly correlated. Traditional univariate analysis lacks the ability to fully, quickly, and accurately explore data’s potential information [8]. Therefore, it is necessary to simplify and classify the data using multivariate statistical methods to obtain detailed information on metabolic differences in yak and cattle-yak samples. 

PCA is an unsupervised clustering method that can be used to outline divergence in volatile components and highlight differences between samples [31]. As shown in Figure 2B, the variance contribution rate of PC1 and PC2 was 37.2% and 14.5% respectively, and the cumulative variance contribution was 51.7%. The two groups of samples were located on both sides of the positive and negative half axes of PC1, without overlap, indicating that there was a clear difference between each metabolite in yak and cattle-yak meat. 

Affected by instrument drift, artifacts, and other experimental variables, the focus of PCA models shifted to system variables that are irrelevant to the scientific question of interest [32]. Therefore, in order to further discover the differences between different samples, the OPLS-DA model was used to classify the samples. OPLS-DA is an analytical method that visualizes and quantifies the extent of differences between samples, based on the correlation between the data [33]. The explanatory ability of the model is expressed by R^2^X and R^2^Y respectively, and the predictive ability is expressed by Q^2^ [12]. The closer R^2^ and Q^2^ are to 1.0, the better the model fits. In Figure 2C, R^2^Y = 0.996, Q^2^ = 0.930, both close to 1, and all the samples were within a 95% confidence interval, indicating that the OPLS-DA model had good interpretation and prediction ability. To determine whether the OPLS-DA was over-fitting, 200 cross-substitution tests were performed on the model. The results are shown in Figure 2D, where the horizontal coordinate is sample retention rate, and point 1.0 is R^2^ and Q^2^ of the original model. It was verified that R^2^ (0.93) and Q^2^ (−0.06) were both less than the retention value 1.0, and the intercept of the Q^2^ regression line of the model with the horizontal coordinate is negative, indicating that the OPLS-DA model does not have over-fitting phenomenon and is stable and reliable.

#### 3.3.3. Differential Metabolite Analysis

VIP value is often used to explain the importance of the variables to the model. VIP > 1 indicates that the characteristic peak is important and is usually used as one of the screening conditions for potential biomarkers [8]. According to OPLS-DA, VIP > 1 and *p* < 0.05 were used as criteria to search for metabolites with significant differences in expression. A total of 75 significant differential metabolites were screened from the two groups of samples (Table 3). Compared with Y, 49 significant differential metabolites such as aspartic acid, glycine, and lysine were up-regulated and 26 significant differential metabolites such as glutamic acid, tyrosine, and glutamine were down-regulated in CY. In order to more intuitively show the relationship between samples and the differences in metabolite expression in different samples, a hierarchical cluster analysis of Y and CY was conducted. From Figure 2E, it can be seen that the metabolite abundances of Y and CY showed significant differences, and all the samples can be divided into two categories. Cluster I included CY1, CY2, CY3, CY4, CY5, and CY6, and Cluster II included Y1, Y2, Y3, Y4, and Y5, which was highly consistent with the results of the PCA model.

Carboxylic acids and derivatives were the most abundant metabolites in Y and CY, among which 17 metabolites such as serine, phenylalanine, and glycine were significantly up-regulated in CY. 10 metabolites such as proline and glutamic acid were significantly up-regulated in Y. The main constituents of flavor precursors include amino acids, peptides, sugars, and lipids [34]. Amino acids are the most abundant non-volatile metabolites that affect meat quality, and are also important flavor substances and flavor precursors in meat, which play a significant part in the complex synthesis of volatile flavor compounds and overall aroma [34,35]. The majority of amino acids are formed by proteins and peptides in the action of enzymes [36]. Different amino acids have different taste thresholds and are classified into umami, sweet, bitter, and tasteless according to their unique flavor [37]. It has been reported that serine and glycine constitute the sweet taste of meat, aspartic acid and glutamic acid constitute the umami taste of meat, and tyrosine and phenylalanine constitute the aromatic taste of meat, which are favored by consumers [38,39].

The contents of serine and glycine in CY were significantly higher than that in Y. Serine and glycine are known as sweet amino acids, and the increase in their contents indicated that cattle-yak was sweeter than yak meat. Aspartic acid and glutamic acid are exclusively umami-tasting to humans, and small peptides containing at least one of these two amino acids above usually exhibit umami flavor [24]. Under the action of transaminase, aspartic acid produces oxaloacetic acid, which further reacts to produce n-butanol, 2-butanone, and other ketones [8]. The content of aspartic acid in CY and glutamic acid in Y was significantly abundant, indicating that the umami taste in the cattle-yak and yak meat was equivalent. Aromatic amino acids such as phenylalanine and tyrosine are precursors of VOCs, which are mainly produced by the oxidative hydrolysis of proteins [8]. Phenylalanine, an essential amino acid in humans and animals, is mainly oxidized to tyrosine by phenylalanine hydroxylase in the body. Moreover, phenylalanine and tyrosine together synthesize neurotransmitters and hormones and are involved in the collective metabolism of sugars and fats [8]. However, the Streker degradation of phenylalanine or the oxidation pathway of linolenic acid produces benzaldehyde, which produces an unpleasant taste and can affect the aroma of meat [8,34]. Compared with Y, the content of phenylalanine in CY was significantly increased, and the content of tyrosine was significantly decreased. Therefore, we estimated that yak meat has a slightly stronger aroma than the cattle-yak meat. Lysine content in CY was significantly abundant. Lysine is an amino acid that produces sugars and ketones, so it can participate in the formation of D-glucose, lipids, and other substances, ultimately producing energy [40]. In addition to amino acids, inosine 5′-monophosphate (IMP), hypoxanthine, and xanthine, which were involved in purine metabolism, were also found to be significantly increased in CY. IMP is an important umami substance in nucleotides, and hypoxanthine produced by hydrolysis of IMP is positively correlated with the sweetness of cooked mutton [35]. The synergistic effect of IMP and amino acids, especially asparagine, glutamine, alanine, and phenylalanine, enhances the umami taste of meat [41]. 

To further analyze the metabolic pathways involved in the differential metabolites in Y and CY, the KEGG database was used to annotate the differential metabolites and conduct enrichment analysis. As shown in Figure 2F, differential metabolites were significantly enriched in the metabolic pathways of arginine biosynthesis and oxidative phosphorylation (*p* < 0.05), among which arginine biosynthesis had the greatest impact in different pathways. Alpha-ketoglutaric acid, aspartic acid, glutamine, ornithine, fumaric acid, and citrulline were significantly enriched in the arginine biosynthesis pathway.

## 4. Conclusions

In this study, the differences in nutrients, VOCs, and non-volatile metabolites between yak and cattle-yak meat were compared. The results showed that the contents of protein, fat, and Fe in yak meat were significantly higher than those in cattle-yak (*p* < 0.05). GC-IMS identified 31 VOCs such as alcohols, ketones, esters, and aldehydes. There were content differences in VOCs such as 2-methylbutanal, (E)-2-pentenal, 2,3-Butanedione, 3-Pentanone, 1-Propanethiol, 3-methylbutanol, 4-Methyl-2-pentanone, and ethyl formate in the two types of meat. GC-MS identified 75 non-volatile metabolites with significant differences, among which serine, glycine, phenylalanine, aspartic acid, lysine, hypoxanthine, xanthine, and IMP were significantly increased in cattle-yak meat. Glutamic acid and tyrosine contents were significantly increased in yak meat, and key metabolic pathways related to the formation of yak and cattle-yak meat flavor were also identified.

## Figures and Tables

**Table 1:** Nutrient composition of yak and cattle-yak meat.

Item (%)	Y	CY
Protein	24.34 ± 0.23 *	22.11 ± 0.17
Fat	1.10 ± 0.03 *	0.36 ± 0.01
P	221.97 ± 5.65	218.55 ± 6.36
Ca	23.38 ± 8.09	18.23 ± 2.95
Fe	20.04 ± 1.04 *	12.84 ± 1.39

The absence of a shoulder label in the same row of data indicates that the difference between the two groups is not significant (*p* > 0.05). * means the difference between the two groups is significant (*p* < 0.05).

**Table 2:** The VOCs of yak and cattle-yak meat identified by GC-IMS.

Volatiles	No	Compounds	CAS ^1^	Formula	RI ^2^	Rt ^3^	Dt ^4^	Intensity	
								Y	CY
Alcohols (5)	1	3-methylbutanol	C123513	C_5_H_12_O	788.2	243.833	1.47812	265.53 ± 49.49	157.87 ± 0.37
	2	1-Propanol, 2-methyl-	C78831	C_4_H_10_O	636.0	135.751	1.18694	692.82 ± 47.55	651.72 ± 14.31
	3	1-Propanethiol	C107039	C_3_H_8_S	636.0	135.751	1.3919	245.22 ± 53.36	151.63 ± 10.94
	4	2-Propanol, 2-methyl-	C75650	C_4_H_10_O	494.6	73.316	1.1322	172.80 ± 75.59	220.23 ± 81.41
	5	2-Propylthiol	C75332	C_3_H_8_S	563.3	103.666	1.13521	69.73 ± 4.64	78.47 ± 0.63
Ketones (5)	6	2-methyl-4,5-dihydro-3(2H)-furanone	C3188009	C_5_H_8_O_2_	819.4	278.786	1.41817	485.91 ± 180	362.77 ± 3.66
	7	2-Pentanone, 3-methyl-	C565617	C_6_H_12_O	749.0	209.743	1.15892	79.77 ± 2.72	93.78 ± 8.70
	8	3-Pentanone	C96220	C_5_H_10_O	700.2	168.748	1.33575	392 ± 29.94	170.95 ± 2.34
	9	2,3-Butanedione	C431038	C_4_H_6_O_2_	581.5	111.712	1.16481	122.66 ± 9.77	74.94 ± 2.24
	10	4-Methyl-2-pentanone	C108101	C_6_H_12_O	699.7	168.296	1.20064	54.45 ± 1.77	106.01 ± 3.31
Esters (5)	11	Ethyl propanoate	C105373	C_5_H_10_O_2_	704.2	172.094	1.45624	86.73 ± 11.81	90.61 ± 0.56
	12	ethyl-2-methyl propanoate	C97621	C_6_H_12_O_2_	704.7	172.513	1.56849	798.93 ± 286.4	598.2 ± 23.09
	13	methyl acetate	C79209	C_3_H_6_O_2_	487.6	70.233	1.18708	116.71 ± 0.89	117.66 ± 2.16
	14	Ethyl formate	C109944	C_3_H_6_O_2_	512.5	81.222	1.05041	1279.73 ± 157.6	341.52 ± 5.18
	15	propyl acetate	C109604	C_5_H_10_O_2_	676.9	153.81	1.17927	44.52 ± 7.11	63.18 ± 1.02
Aldehydes (3)	16	(E)-2-pentenal	C1576870	C_5_H_8_O	752.1	212.332	1.33875	520.99 ± 146.18	439.67 ± 10.75
	17	2-methylbutanal	C96173	C_5_H_10_O	688.8	159.123	1.16947	235.71 ± 48.04	246.5 ± 1.46
	18	Isopentanal	C590863	C_5_H_10_O	614.7	126.38	1.17625	92.06 ± 3.84	90.59 ± 5.04
Furan (2)	19	Tetrahydrofuran	C109999	C_4_H_8_O	584.5	113.047	1.25448	2812.02 ± 521.38	2294.83 ± 167.86
	20	2,5-dimethylfuran	C625865	C_6_H_8_O	673.1	152.158	1.3362	1017.06 ± 8.71	364.01 ± 30.63
Alkanes (2)	21	2,3,4-Trimethylpentane	C565753	C_8_H_18_	751.1	211.469	1.71339	171.39 ± 129.01	83.53 ± 5.33
	22	1,1-Diethoxyethane	C105577	C_6_H_14_O_2_	700.7	169.162	1.10715	171.25 ± 29.27	76.18 ± 4.73
Amines (1)	23	1-Propanamine	C107108	C_3_H_9_N	487.5	70.21	1.07889	144.68 ± 26.94	187.80 ± 6.28
Acids (1)	24	Acetic acid	C64197	C_2_H_4_O_2_	576.2	109.375	1.08213	1639.96 ± 126.12	1745.76 ± 29.06
Thiazoles (1)	25	thiazole	C288471	C_3_H_3_NS	712.5	179.104	1.27281	588.83 ± 43.15	687.71 ± 7.26
Pyrazines (1)	26	2,3-dimethylpyrazine	C5910894	C_6_H_8_N_2_	903.9	378.899	1.48561	762.49 ± 115.69	648.43 ± 6.99
Others (5)	27	Allyl cyanide	C109751	C_4_H_5_N	647.9	141.018	1.12732	374.48 ± 7.90	276.28 ± 3.04
	28	Pyrrolidine	C123751	C_4_H_9_N	694.5	163.927	1.25981	141.21 ± 25.2	280.02 ± 6.93
	29	Carbon disulfide	C75150	CS_2_	531	89.417	1.12637	2304.87 ± 181.67	1374.04 ± 78.92
	30	dimethyl disulfide	C624920	C_2_H_6_S_2_	787.9	243.401	1.15443	717.05 ± 80.74	644.24 ± 8.18
	31	Ethylsulfide	C352932	C_4_H_10_S	683.2	156.598	1.06786	1293.72 ± 59.26	1108.41 ± 32.41

^1^ represents the registration number of chemical substances by Chemical Abstracts Service. ^2^ represents the retention index calculated using n-ketones C4–C9 as external standard on FS-SE-54-CB column. ^3^ represents the retention time in the capillary GC column. ^4^ represents the drift time in the drift tube.

**Table 3:** The differential non-volatile metabolites of yak and cattle-yak meat identified by GC-TOF-MS.

Number	Metabolites	VIP ^1^	*p*-Value	log_2__FC(CY/Y) ^2^	Trend
1	Glucose-1-phosphate	1.56002	3.87 × 10^−6^	1.08187	Up
2	pantothenic acid	1.49677	1.10 × 10^−4^	−1.09597	Down
3	fructose	1.49492	1.06 × 10^−4^	1.39380	Up
4	Palatinose	1.45582	1.93 × 10^−4^	3.02793	Up
5	ribulose-5-phosphate	1.34827	2.72 × 10^−3^	9.48115	Up
6	xylitol	1.23574	5.28 × 10^−3^	0.57462	Up
7	1,5-Anhydroglucitol	1.20417	9.13 × 10^−3^	−1.17174	Down
8	Erythrose	1.20173	9.20 × 10^−3^	0.21801	Up
9	oxamic acid	1.57446	2.94 × 10^−6^	3.22510	Up
10	sarcosine	1.52540	5.60 × 10^−5^	−0.74482	Down
11	canavanine	1.52044	4.37 × 10^−5^	2.47246	Up
12	proline	1.51984	3.93 × 10^−5^	−1.55970	Down
13	1-Aminocyclopropanecarboxylic acid	1.50193	1.35 × 10^−4^	1.27751	Up
14	N-Ethylglycine	1.45510	4.18 × 10^−4^	0.34896	Up
15	serine	1.44050	2.65 × 10^−4^	0.88418	Up
16	3-Aminoisobutyric acid	1.38257	1.84 × 10^−3^	0.45469	Up
17	glutamic acid	1.42205	6.80 × 10^−4^	−1.42754	Down
18	tyrosine	1.34875	2.33 × 10^−3^	−0.77930	Down
19	N-Methyl-DL-alanine	1.32256	4.01 × 10^−3^	−0.70170	Down
20	beta-Glutamic acid	1.31557	3.14 × 10^−3^	2.24210	Up
21	phenylalanine	1.31458	3.45 × 10^−3^	−0.35167	Down
22	Aminomalonic acid	1.33658	1.87 × 10^−3^	1.16951	Up
23	histidine	1.29907	6.14 × 10^−3^	−0.93743	Down
24	glutamine	1.24694	9.32 × 10^−3^	−0.63591	Down
25	glycine	1.24456	5.73 × 10^−3^	0.75665	Up
26	glycocyamine	1.24334	9.08 × 10^−3^	0.36231	Up
27	aspartic acid	1.24292	5.14 × 10^−3^	1.48157	Up
28	succinic acid	1.25651	3.74 × 10^−3^	1.39523	Up
29	asparagine	1.20055	1.18 × 10^−2^	0.40159	Up
30	malonic acid	1.19689	1.27 × 10^−2^	0.31814	Up
31	lysine	1.15707	1.08 × 10^−2^	0.88380	Up
32	citrulline	1.14803	1.85 × 10^−2^	−0.19373	Down
33	fumaric acid	1.14731	1.79 × 10^−2^	−0.65897	Down
34	norvaline	1.11270	2.97 × 10^−2^	0.73737	Up
35	ornithine	1.00617	4.11 × 10^−2^	0.89192	Up
36	heptadecanoic acid	1.34592	2.04 × 10^−3^	−0.59299	Down
37	Myristic Acid	1.24276	9.90 × 10^−3^	−0.28060	Down
38	Linoleic acid methyl ester	1.12423	2.23 × 10^−2^	−2.49612	Down
39	benzoic acid	1.42995	3.78 × 10^−4^	0.73302	Up
40	Hippuric acid	1.42225	3.81 × 10^−4^	1.31543	Up
41	terephthalic acid	1.29725	2.65 × 10^−3^	0.95815	Up
42	phthalic acid	1.27977	6.66 × 10^−3^	−1.18342	Down
43	hypoxanthine	1.57578	1.87 × 10^−6^	2.55151	Up
44	xanthine	1.33276	2.35 × 10^−3^	2.19248	Up
45	inosine	1.48286	6.92 × 10^−5^	2.64073	Up
46	2-Deoxyuridine	1.31110	4.24 × 10^−3^	−0.29888	Down
47	Menthone	1.20062	1.19 × 10^−2^	0.86945	Up
48	Phytol	1.12695	2.42 × 10^−2^	−0.32129	Down
49	Methyl Phosphate	1.43979	5.12 × 10^−4^	−1.97551	Down
50	O-Phosphorylethanolamine	1.39927	1.31 × 10^−3^	0.52850	Up
51	spermine	1.07625	2.05 × 10^−2^	2.55276	Up
52	Carnitine	1.43996	6.95 × 10^−4^	2.41044	Up
53	4-Hydroxymandelonitrile	1.42021	4.45 × 10^−4^	1.53866	Up
54	Maleimide	1.38952	1.86 × 10^−3^	−0.25359	Down
55	1-Monopalmitin	1.26962	4.04 × 10^−3^	0.72400	Up
56	ascorbate	1.25331	7.48 × 10^−3^	0.94801	Up
57	phosphate	1.17981	1.05 × 10^−2^	0.26563	Up
58	pyrophosphate	1.29196	4.78 × 10^−3^	−3.02879	Down
59	piceatannol	1.17890	1.45 × 10^−2^	4.32480	Up
60	3-hydroxybutyric acid	1.10009	3.38 × 10^−2^	−0.96013	Down
61	thymine	1.08981	3.59 × 10^−2^	2.11162	Up
62	alpha-ketoglutaric acid	1.08008	2.55 × 10^−2^	−10.37526	Down
63	conduritol b epoxide	1.53321	1.71 × 10^−5^	1.29911	Up
64	D-Talose	1.50914	3.82 × 10^−5^	1.37435	Up
65	inosine 5′-monophosphate	1.50004	4.25 × 10^−5^	2.69415	Up
66	D-Talose	1.49641	5.52 × 10^−5^	1.42748	Up
67	Adenosine 5-monophosphate	1.40342	6.68 × 10^−4^	1.35875	Up
68	(2R)-2-amino-3-phosphonopropanoic acid	1.38789	7.54 × 10^−4^	1.60379	Up
69	Cholestane-3,5,6-triol	1.38680	1.77 × 10^−3^	−0.73503	Down
70	Purine riboside	1.37204	9.81 × 10^−4^	1.59359	Up
71	leucine	1.32632	1.89 × 10^−3^	16.56495	Up
72	8-Aminocaprylic acid	1.32304	4.16 × 10^−3^	−0.86835	Down
73	4-hydroxybutyrate	1.27964	3.72 × 10^−3^	0.92631	Up
74	5-Dihydrocortisol	1.21005	7.33 × 10^−3^	0.41992	Up
75	alpha-D-glucosamine 1-phosphate	1.07065	4.32 × 10^−2^	−0.52420	Down

^1^ represents the variable importance in the projection of OPLS-DA. ^2^ represents the log_2_ value of the fold change.

## Data Availability

The original contributions presented in the study are included in the article and supplementary materials, further inquiries can be directed to the corresponding authors.

## Supplementary Materials

The following supporting information can be downloaded at: https://www.mdpi.com/article/10.3390/foods13152364/s1, Figure S1: The total ion chromatography of quality control samples; Figure S2: The total ion chromatography of yak and cattle-yak meat samples; Table S1: The non-volatile metabolites of yak and cattle-yak meat identified by GC-TOF-MS.
