# Supplementary material for: The Combined Analysis of GC-IMS and GC-MS Reveals the Differences in Volatile Flavor Compounds between Yak and Cattle-Yak Meat
Source: Foods. 2024 Jul 26;13(15):2364. doi: 10.3390/foods13152364 (PMC11311445; doi:10.3390/foods13152364)
Supplement: Supplementary file 1 [file foods-13-02364-s001.zip › foods-3111566-supplementary/Supplementary Table S1.pdf]

Table S1. The non-volatile metabolites of yak and cattle-yak meat identified by GC-TOF-MS

No	Metabolites	Y[1]	CY[2]
Carboxylic acids and derivatives (41)			
1	alanine 1	22423303.46	22206662.62
2	glycine 1	1664383.224	1304394.931
3	oxalic acid	9	18585.6342
4	sarcosine	197492.4116	117852.4067
5	N-Methyl-DL-alanine	133546.4308	82110.7027
6	malonic acid 1	226595.0908	282500.6638
7	valine	1382165.152	1171946.1
8	1-Aminocyclopropanecarboxylic acid	43366.1468	105128.3393
9	norvaline	45834.9614	76413.1825
10	Isoleucine	480893.49	452496.4925
11	proline	2029500.808	688451.4452
12	glycine 2	1915121.024	3235711.977
13	succinic acid	1261374.803	3317803.725
14	fumaric acid	668648.3272	423475.4748
15	serine 1	646611.9534	1193461.089
16	cycloleucine 1	34241.0922	29174.7128
17	threonine 1	195884.2836	192301.4398
18	N-Ethylglycine 1	211310.4856	269133.1915
19	beta-Alanine 2	80309.476	107925.1335
20	3-Aminoisobutyric acid 1	197749.924	271013.6825
21	Aminomalonic acid	89812.3682	202019.1565

22	aspartic acid 1	14303.8224	39943.703
23	oxoproline	22053339.38	19483035.07
24	trans-4-hydroxy-L-proline 2	6361.7664	3394.033
25	L-cysteine	68482.4364	71288.759
26	creatine	34864572.54	37053592.07
27	asparagine 2	20871.552	27570.4787
28	beta-Glutamic acid 1	79088.2394	374155.3053
29	ornithine	75120.2116	81650.6515
30	glutamic acid	167680.905	62337.8028
31	phenylalanine 1	261373.2644	204832.3453
32	5-Aminovaleric acid 1	87703.9972	121617.6233
33	citrulline 2	145939.638	127600.9288
34	glutamine 1	410562.428	264211.0278
35	citric acid	9705.1312	3634.8963
36	ornithine 1	73937.761	137202.1975
37	histidine 2	187277.8276	97789.5367
38	lysine	92208.0896	170145.6018
39	canavanine 1	2301.9232	12775.3898
40	tyrosine 1	397036.2588	231333.6822
41	glycocyamine 1	79309.4566	101950.7317
Organooxygen compounds (21)			
42	Lactamide 1	31150.0924	27871.4415
43	glycerol	762038.3152	905487.2112
44	D-Glyceric acid	51981.3094	47625.6082
45	Erythrose 1	380904.0912	443040.842

46	Threonic acid	10490.6724	14512.118
47	allose 1	91186.9734	137318.047
48	xylitol	8257.689	12298.0348
49	Glucose-1-phosphate	117984.9806	249748.0918
50	1,5-Anhydroglucitol	17148.7066	7612.0482
51	fructose 1	329152.9702	864912.4373
52	sorbitol	71367.1076	45021.0108
53	pantothenic acid	23879.5062	11171.3763
54	mucic acid	4562.0544	1682.6607
55	myo-inositol	1929011.068	3234355.972
56	ribulose-5-phosphate 2	9	6432.0902
57	Fructose 2,6-biphosphate degr prod 2	3341216.69	3812241.813
58	glucose-6-phosphate 1	2531769.394	2318214.158
59	6-phosphogluconic acid	187720.4384	171398.0663
60	maltose	22813.114	22009.0833
61	Palatinose	2554.3892	20834.6123
62	fructose-6-phosphate	1290114.172	1177991.645
Fatty Acyls (12)			
63	Pelargonic acid	24189.9236	25895.4312
64	Myristic Acid	34851.875	28691.7005
65	1-Hexadecanol	118308.8534	109823.5128
66	palmitic acid	2703403.6	2351280.53
67	Linoleic acid methyl ester	8073.1988	1430.9943
68	heptadecanoic acid	13753.7508	9118.3012
69	Octadecanol	4737.7542	2723.3115

70	oleic acid	42700.0814	42106.4447
71	stearic acid	1678469.08	1443237.49
72	arachidonic acid	9	9
73	Arachidic acid	8112.9276	6989.7985
74	Turanose 2	7321.904	14674.0413
Benzene and substituted derivatives (6)			
75	benzoic acid	28080.0242	46672.1703
76	phthalic acid	511439.3312	225189.4822
77	2,3-dihydroxybenzoic acid	9	1583.0138
78	terephthalic acid	84042.459	163279.0038
79	Hippuric acid 2	25317.0228	63008.1677
80	3-Hydroxyanthranilic acid	4413.305	3298.7022
Hydroxy acids and derivatives (5)			
81	glycolic acid	202886.068	182361.8775
82	2-hydroxybutanoic acid	21761.0866	20914.8637
83	3-Hydroxypropionic acid 1	63694.4122	61199.887
84	3-hydroxybutyric acid	457336.6794	235076.1617
85	L-Malic acid	4553866.644	4812711.148
Prenol lipids (4)			
86	Thymol	13668.8652	15214.973
87	Menthone 1	62533.9594	114247.2647
88	Phytol	62935.7486	50371.0017
89	squalene	193512.0206	209360.1675
Imidazopyrimidines (3)			
90	hypoxanthine 1	63276.913	370959.3665

91	xanthine	3922.7266	17930.361
92	uric acid	10223.7156	6796.8718
Organic phosphoric acids and derivatives (3)			
93	Methyl Phosphate	419618.8704	106700.653
94	Phenylphosphoric acid	102559.5954	118201.8758
95	O-Phosphorylethanolamine	107212.0062	154645.2197
Diazines (2)			
96	uracil	22882.0686	29042.682
97	thymine	2278.4718	9846.9927
Glycerolipids (2)			
98	1-Monopalmitin	51106.1428	84415.0002
99	Monostearin	19127.1708	18759.8563
Indoles and derivatives (2)			
100	tryptophan 1	84307.9156	71522.4778
101	5-Hydroxyindole-3-acetic acid 1	14555.2362	13376.299
Keto acids and derivatives (2)			
102	Pyruvic acid	145884.9574	84252.1055
103	alpha-ketoglutaric acid	11953.8564	9
Non-metal oxoanionic compounds (2)			
104	sulfuric acid	16715.2996	14608.7563
105	pyrophosphate 3	58016.4664	7108.7518
Purine nucleosides (2)			
106	inosine	54583.1264	340406.656
107	xanthosine	9	9
Pyridines and derivatives (2)			

108	2,3-Dihydroxypyridine	9	20423.244
109	2-hydroxypyridine	3947970.802	4472723.888
Pyrimidine nucleosides (2)			
110	2-Deoxyuridine	181675.3616	147681.2403
111	uridine 2	9	9
Stilbenes (2)			
112	Benzoin 2	103962.2992	71214.139
113	piceatannol 2	371.4868	7444.5382
Others (9)			
114	ascorbate	28984.2324	55916.7037
115	taurine	931416.873	852538.3168
116	spermine 3	49026.1864	287664.9272
117	conduritol b epoxide 2	81617.563	200842.7002
118	4-Hydroxymandelonitrile 1	63050.7966	183178.0427
119	7,8-Dimethylalloxazine	6859.5616	2325.0247
120	Maleimide	101244.5828	84924.5478
121	hexadecane	3689577.132	4245918.642
122	cyclohexylsulfamic acid 1	131086.4434	110887.5193

The values in columns 1 and 2 represent the integral quantitative values of each non-volatile metabolite in yak and cattle-yak samples, respectively.
