# Supplementary figures and images for: The Combined Analysis of GC-IMS and GC-MS Reveals the Differences in Volatile Flavor Compounds between Yak and Cattle-Yak Meat
Source: Foods. 2024 Jul 26;13(15):2364. doi: 10.3390/foods13152364 (PMC11311445; doi:10.3390/foods13152364)

**Figure S1.** The total ion chromatography of quality control samples

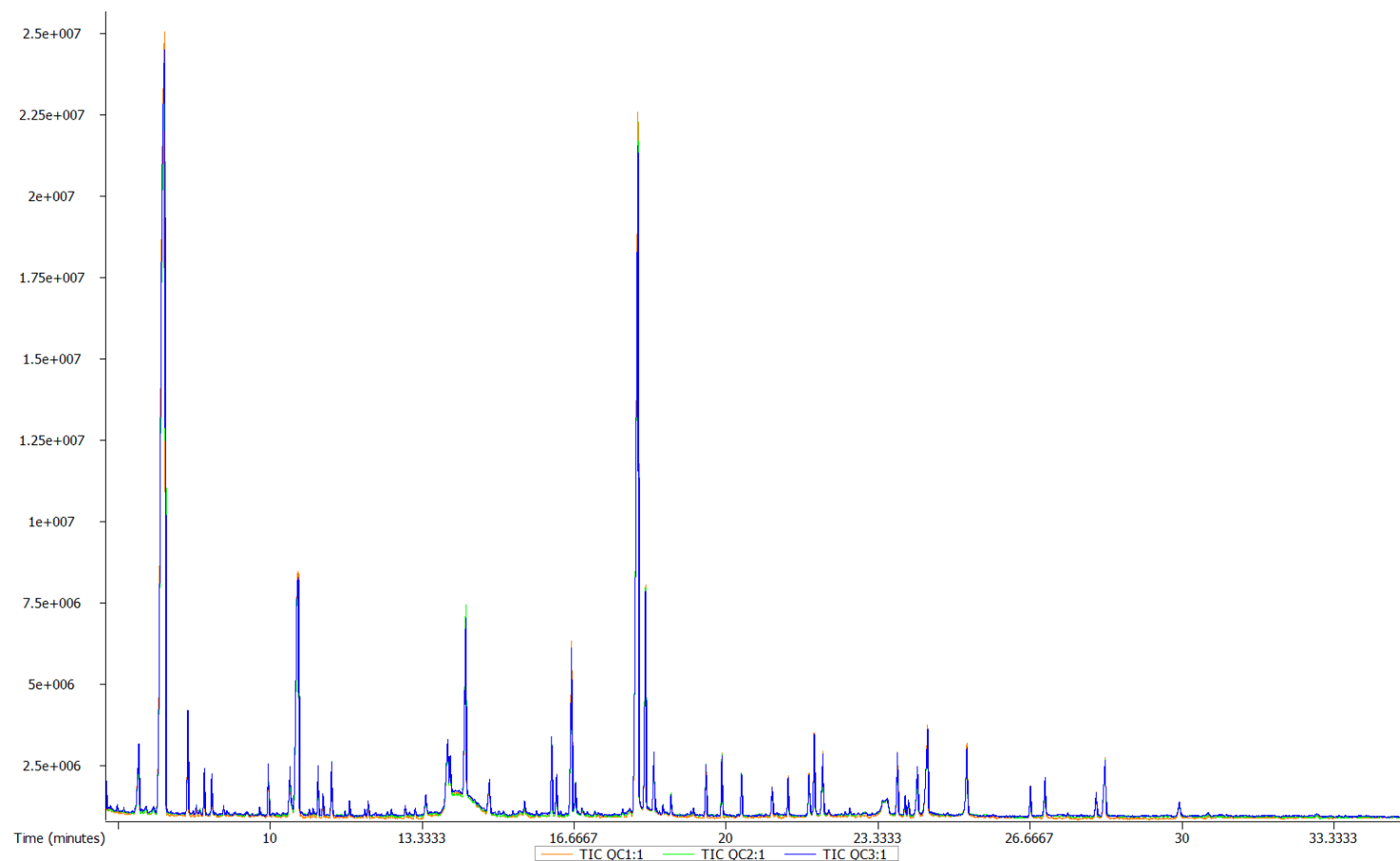

Supplement: Supplementary file 1 [file foods-13-02364-s001.zip › foods-3111566-supplementary/Supplementary Figure S1.pdf]

Figure S2. The total ion chromatography of yak and cattle-yak meat samples

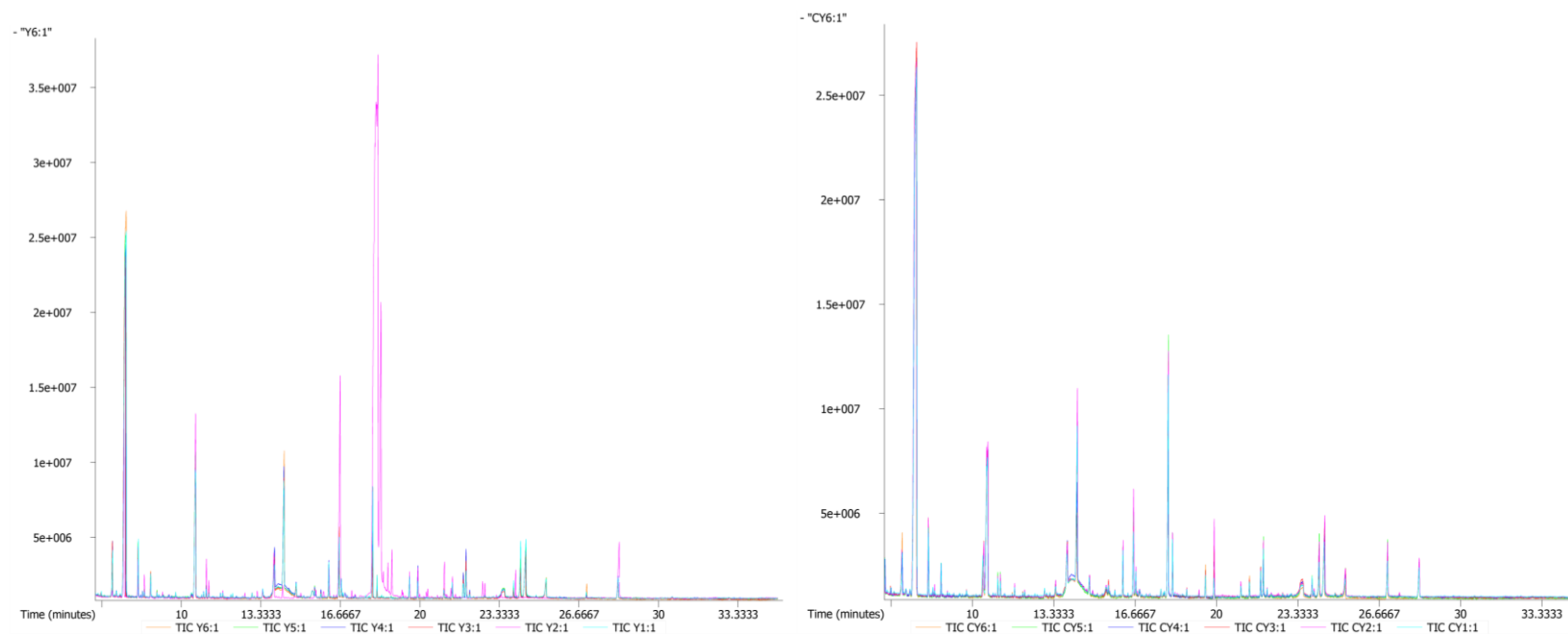

Supplement: Supplementary file 1 [file foods-13-02364-s001.zip › foods-3111566-supplementary/Supplementary Figure S2.pdf]
